# NIR-II Aggregation-Induced Emission Luminogens for Tumor Phototheranostics

**DOI:** 10.3390/bios12010046

**Published:** 2022-01-17

**Authors:** Yonghong Tan, Peiying Liu, Danxia Li, Dong Wang, Ben Zhong Tang

**Affiliations:** 1 Center for AIE Research, Shenzhen Key Laboratory of Polymer Science and Technology, Guangdong Research Center for Interfacial Engineering of Functional Materials, College of Materials Science and Engineering, Shenzhen University, Shenzhen 518060, China; 2110343013@email.szu.edu.cn (Y.T.); 2110343005@email.szu.edu.cn (P.L.); 2110343102@email.szu.edu.cn (D.L.); 2 Shenzhen Institute of Aggregate Science and Technology, School of Science and Engineering, The Chinese University of Hong Kong, Shenzhen 518172, China; tangbenz@cuhk.edu.cn

**Keywords:** aggregation-induced emission, NIR-II emission, phototheranostics, cancer treatment

## Abstract

As an emerging and powerful material, aggregation-induced emission luminogens (AIEgens), which could simultaneously provide a precise diagnosis and efficient therapeutics, have exhibited significant superiorities in the field of phototheranostics. Of particular interest is phototheranostics based on AIEgens with the emission in the range of second near-infrared (NIR-II) range (1000–1700 nm), which has promoted the feasibility of their clinical applications by virtue of numerous preponderances benefiting from the extremely long wavelength. In this minireview, we summarize the latest advances in the field of phototheranostics based on NIR-II AIEgens during the past 3 years, including the strategies of constructing NIR-II AIEgens and their applications in different theranostic modalities (FLI-guided PTT, PAI-guided PTT, and multimodal imaging-guided PDT–PTT synergistic therapy); in addition, a brief conclusion of perspectives and challenges in the field of phototheranostics is given at the end.

## 1. Introduction

Cancer, one of the deadliest diseases in recent decades, has remained a global health concern due to its growing morbidity rate, developing relapse rate, and low survival rate [1,2,3]. Traditional cancer diagnostic methods, including magnetic resonance imaging (MRI), positron emission tomography (PET), and computed tomography (CT), exhibit some respective and collective drawbacks such as insufficient sensitivity and specificity, high cost, and cumbersome instrumentation [4,5]. Those conventional therapeutic methods toward cancers (such as surgical removal, chemotherapy, and radiotherapy) commonly cause side effects, systematic toxicity, unavoidable invasion, and high relapse rate [6]. In general, conventional protocols for cancer diagnostics and therapeutics are individually conducted, which could result in the inefficiency of the curing process and the inaccuracy of treatments. Given the circumstances, tremendous efforts have been made to explore more effective approaches for cancers treatment, among which phototheranostics is a significant advancement that enables the ingenious integration of precise photodiagnostic imaging with phototherapeutic intervention in a single system within spatial colocalization [7,8,9]. This inspiration stirs researchers’ increasing interest in both fundamental studies and clinical trials, mainly on account of its intrinsic advantages, such as simultaneously accurate diagnosis with in situ effective therapy, improved pharmacokinetics, maximized efficacy, optimized drug safety, elevated sensitivity, and specificity in comparison with traditional cancer treatments.

Various modalities are involved in phototheranostic systems, including therapeutic methods such as photodynamic therapy (PDT) and photothermal therapy (PTT), and diagnostic technologies such as photothermal imaging (PTI), photoacoustic imaging (PAI), and fluorescence imaging (FLI). As an emerging strategy for cancer treatments via generating reactive oxygen species (ROS) with the assistance of light, tissue oxygen, and photosensitizer (PS), PDT has a remarkable light-controllable ability, specific spatiotemporal selectivity, and minimized invasiveness [10]. PTT is another effective light-triggered cancer therapy modality, which affords excellent tumor suppression by sufficient thermal production upon photoirradiation [11]. Moreover, the thermal signal generated during PTT can be detected by thermal imaging systems for PTI, providing images with great temperature sensitivity for tumor detection. Apart from that, the generated thermal signal gives rise to the rapid thermoelastic expansion of tissue, based on which the light-triggered diagnostic protocol, PAI, can be established, sufficing to provide imaging with high penetration depth and portray clear tumor profiles [12]. Among all photodiagnostic modalities, FLI has aroused intense interest on account of its simple operation, high sensitivity, noninvasive features, and preferable biosafety especially organic fluorophores [13,14,15]. However, FLI generally suffers from some drawbacks in terms of tissue penetration and spatial resolution, which hinders its practical utilization. Moreover, conventional organic fluorophores are ordinarily hydrophobic, which inherently form aggregates in a physiological environment that is generally composed of water, causing local concentration increasing and fluorescence quenching, which is the notorious aggregation-caused quenching (ACQ) effect, consequentially leading to unsatisfactory imaging outcomes.

Fortunately, aggregation-induced emission (AIE), a unique phenomenon discovered in 2001 by Tang, has solved this predicament, which is shown in some twisted-structure molecules with propeller-shaped conformation, tetraphenylethene (TPE) derivatives, for instance. The emissions of AIEgens demonstrate a low intensity in a single molecular state but are enhanced in aggregated state, exhibiting completely contrary features to ACQ [16,17,18]. Numerous endeavors have been made to explore the mechanism of AIE phenomenon, and the restriction of intramolecular motion (RIM) that includes restriction of intramolecular rotation (RIR) and restriction of intramolecular vibration (RIV) has been widely approved, according to which the twisted structure and the sufficient structural rotors and/or vibrators jointly endow AIE luminogens (AIEgens) with the distinct characteristics [7,19]. Due to the structural superiorities, most of the excited-state energy of AIEgens can be consumed through the nonradiative decay pathway, resulting from vigorous intramolecular motions in the single molecular state, consequently promoting photothermal conversion. On the contrary, the intramolecular motions can be suppressed in an aggregated state; thus, the radiative decay pathway is in the dominant position, consequently boosting fluorescent emission. In addition, AIEgens have been recognized to possess many intrinsic advantages including good biocompatibility, large Stokes shift, excellent tolerance for high concentration, turn-on feature, high photobleaching threshold and outperformed photosensitizing features, which all allow the great potential for efficient phototheranostics.

On the other hand, enthused by the remained shortcomings of fluorescence imaging with visible (400–680 nm) and first near-infrared region (NIR-I, 700–900 nm), including low tissue penetration, unsatisfactory spatial resolution, etc., researchers pay attention to develop fluorescent materials with emission in the range of second near-infrared (NIR-II) window to overcome these drawbacks [20,21,22,23,24,25]. NIR-II fluorophores possess the capability of surmounting the inherent deficiencies of conventional FLI, by virtue of its remarkable features including deep penetration, reduced tissue scattering, minimal damage, and high spatial resolution endowed by the extremely long wavelength [26,27,28]. The combination of the advantages of both NIR-II fluorophores and AIEgens unprecedentedly complemented each other with excellent imaging and extraordinary therapy, thus allowing a better application in the clinical field and accelerating the progression of contemporary precision medicine [29,30]. Witnessing the rapid development and great significance of theranostic researches based on NIR-II AIEgens, it is crucial to publish a comprehensive review article to systematically generalize the merits of NIR-II AIEgens in cancer theranostics and to provide an integrated picture of this area through the introduction of basic concepts and recent trends as well as novel perspectives.

In this minireview, we summarize the recent advances of NIR-II AIEgens (Table 1) in the cancer theranostic field during the past three years. In the first section, the breakthroughs in photothermal therapy under the guidance of FLI generated by NIR-II AIEgens are primarily elaborated. In the second section, up-to-date signs of progress observed in photothermal therapy under the guidance of PAI are presented subsequently, as well as the design strategies and mechanistic insights of theranostics. In the third section, boosted multimodality theranostics systems based on NIR-II AIEgens are listed, and the complex construction and the favorable superiorities of the systems are discussed in detail as well. The existing limitations and novel perspectives in this field conclude the study. We expect that this review will provide valuable insights into NIR-II AIEgens-based theranostics and serve as an inspiration for developing integrated systems of diagnostics and therapeutics, thereby stimulating more studies at this research frontier.

## 2. NIR-II FLI-Guided PTT

**Figure 1:**
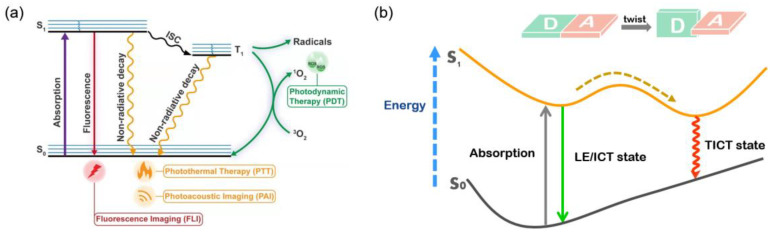
(**a**) Schematic illustration of Jablonski diagram. Reprinted with permission from Ref. [35]. Copyright 2021, Light Publishing Group; (**b**) the illustration of TICT mechanism.

Nowadays, FLI in NIR-II has become a momentous facility for cancer diagnosis owing to its prominent merits for in vivo monitoring and visualizing of lesions [31,32]. Additionally, the combination of NIR-II FLI and PTT could provide unlimited prospects to construct outstanding theranostic systems [33,34]. As illustrated in Figure 1a [35], when a fluorophore absorbs photons or other energy, it can be promoted to the excited states (S_n_) from the ground state (S_0_) and transfers back to the ground state via either radiative or nonradiative decay. Nevertheless, it is not difficult to find that these two modes of energy dissipation are in competition with each other since energy is conserved. As a result, the strategy to keep the equilibrium between fluorescence (radiative decay) and photothermal effect (nonradiative decay) is the focus of FLI-guided PTT.

Fortunately, AIEgens exhibit free-moving molecular rotators or vibrators in their structure, which are ideal agents to keep the equilibrium between fluorescence and photothermal effect [36]. Using reverse thinking of the AIE process, researchers devised numerous strategies to maximize molecular motion in the aggregated state of AIEgens to exhibit superior heat transitions without compromising FLI [37]. In addition, it was found that twisted intramolecular charge transfer (TICT) states in AIEgens typically abate the fluorescence signals but enhance their photothermal capability, which quickly sparked strong interest among researchers [38,39,40].

AIEgens with long emission wavelengths generally have powerful electron donor (D)–acceptor (A) strength and, therefore, are candidates to modulate TICT formations, since increasing the D–A effect can achieve red-shifted emission and stabilize the TICT state by facilitating charge separation (Figure 1b) [41,42]. When AIEgen is under unbound and free rotating conditions, nonradiative decay would dominate the excited-state energy consumption. In contrast, upon reaching an aggregated state, the physical constraints disable TICT formations; thus, the equilibrium moves to the radiative decay pathway accompanied withbright fluorescence [43,44]. Therefore, tailoring intramolecular motion is a feasible strategy to realize a subtle balance between fluorescence and photothermal effect [45,46].

**Figure 2:**
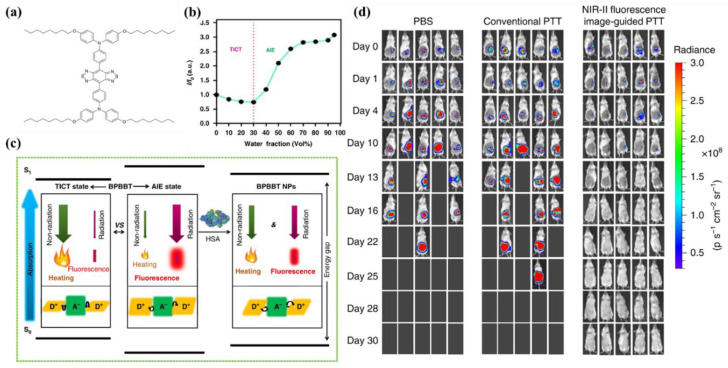
(**a**) Chemical structure of BPBBT; (**b**) plot of fluorescence intensity ratio of BPBBT (10 µM) in water/THF mixture; (**c**) the illustration of has-altering radiative decay and nonradiative decay of BPBBT; (**d**) the in vivo fluorescence imaging of BALB/c mice bearing orthotopic CT26 colon cancer before or after different treatment (n = 5). Reprinted with permission from Ref. [47]. Copyright 2019, Nature Publishing Group.

Lu et al. [47] reported a strategy inspired by the theory of RIR to tailor the equilibrium of fluorescence and photothermal efficiency. They combined NIR-II AIEgen (BPBBT, Figure 2a) with human serum albumin (HSA), in order to restrict the intramolecular rotation of BPPBT. Fluorescence emission spectra demonstrated that the fluorescence intensity of BPBBT decreased at a fraction value of water (*f*_w_) below 30% but increased when further raising *f*_w_. This phenomenon could be explained by the fact that BPBBT transitioned from LE state to TICT state when the polarity of solvent elevated, then the increase in poor solvent contributed to forming the aggregated state of BPBBT, which prevented TICT formations and enhanced the fluorescence emission (Figure 2b). It was found that with the enhancement of the HSA ratio in BPBBT/HSA complexes (BPBBT NPs), the photothermal effect was further increased. The energy difference between S_1_ and S_0_ of BPBBT at NPs state was determined to be narrower than at the AIE state but broader than at the TICT state (Figure 2c), which evinced that the addition of HSA successfully altered LE and TICT state by raising the dihedral angles to provide a chance for the equilibrium to move to the TICT state. In vivo biological imaging showed that fluorescence signal was detected in orthotopic and metastatic tumors accurately and reached a maximum at 30 h postinjection. Notably, NIR-II imaging-guided PTT based on BPBBT NPs could precisely distinguish lesions with dimensions as small as 0.5 mm × 0.3 mm and completely cure tumor-bearing mice with the optimized laser doses (5 out of 5) without recurrence in 30 days (Figure 2d). Additionally, compared with HSA/indocyanine green (ICG) complexes that were applied to NIR-I imaging-guided PTT, BPBBT NPs provided more accurate and sensitive imaging and exhibited a higher photothermal conversion effect and better photostability, which dramatically enhanced the efficiency of PTT and prevented from omitting small lesions. Above all, the BPBBT NPs displayed great potential in NIR-II FLI-guided PTT, particularly for colon cancer theranostics.

**Figure 3:**
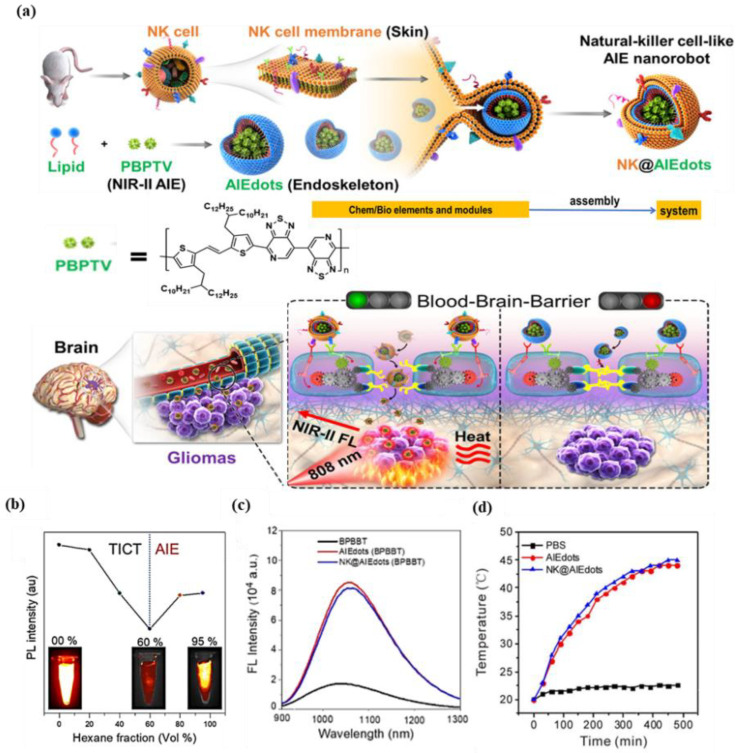
(**a**) Schematic illustration of the preparation and assembly process of NK-cell-mimic AIE nanoparticles (NK@ AIEdots); (**b**) plot of fluorescence intensity of PBPTV in dichloromethane/hexane mixtures; (**c**) fluorescence spectra of BPBBT, AIEdots (BPBBT), NK@AIEdots (BPBBT) in water; (**d**) photothermal effect of PBS, AIEdots, and NK@AIEdots; Reprinted with permission from Ref. [49]. Copyright 2020, American Chemical Society.

Thus far, there are few reliable strategies available for through-skull imaging and therapy, because blood–brain barrier (BBB) is an intractable obstruction for various nanoparticles/macromolecule into the brain [36,48]. Tang group [49] developed the natural killer (NK) cell-mimic nanorobots with highly bright NIR-II fluorescence, named NK@AIEdots, to construct smart and safe multifunctional nanoplatforms for BBB-crossing and brain-tumor-targeting through-skull imaging and therapy (Figure 3a). NK@AIEdots wrap a natural kill cell membrane on a reported highly bright NIR-II AIE-active conjugated polymer nanoendoskeleton, PBPTV. The inspiration for this strategy came from the remarkable properties of NK cells whose membrane can form a “green channel” to help NK@AIEdots realize the BBB crossing [50]. PBPTV is the low-bandgap-conjugated polymer with a high quantum yield (QY) up to 8.6%, which is constructed by using a strong and twisted dual-electron acceptor (BPT). BPT results in long-wavelength absorption and also promotes the intramolecular motion, thus tailoring the equilibrium of the TICT and AIE states (Figure 3b). It was observed that NK@AIEdots displayed bright and long emissions at the NIR-II region, as well as having outstanding photothermal effects (Figure 3c,d). Meanwhile, NK@AIEdots could successfully pass through the BBB and spontaneously accumulate in glioma cells in vivo owing to tumor-targeting proteins of the NK cell membrane, as well as lit up the glioma as intense NIR-II fluorescence even at 48 h postinjection. Moreover, upon NIR light irradiation, NK@AIEdots could effectively inhibit the growth of brain tumor cells with less weight loss in mice, compared with the two control groups. In brief, the NK@AIEdots-based theranostics platform successfully applied to the BBB-crossing and brain-tumor-targeting through-skull FLI-guided PTT.

Xu et al. [51] developed a phototheranostics platform based on a single molecule with intense fluorescence in the NIR-II region. In this study, they prepared DTPA–BBTD by means of changing the electron-withdrawing substituent groups in the molecular backbone of a D–A–D-type AIEgen, which was previously designed by the same group. Compared with the original molecule, DTPA–BBTD exhibited more twisted conformation and smaller bandgap, both of which enhanced TICT and photothermal effect. As-prepared DTPA–BBTD-based AIE dots could produce bright and well-distributed fluorescence signals through the liver/spleen/head regions even in the blood vessels. Researchers found that the presented AIE dots could efficiently suppress tumor growth upon laser irradiation. Meanwhile, pathological examinations also suggested the AIE dots exhibited excellent biocompatibility with negligible cytotoxicity to normal tissues. In summary, a feasible tactic was conducted to design an AIE agent with PPT effect, which is a potential candidate for NIR-II FLI and NIR-I PAI-guided high-efficiency PTT.

## 3. PAI-Guided PTT Based on NIR-II Fluorophores

With the explorations of the potential of NIR-II phototheranostics, the phenomenon that the brightness of organic fluorophores including AIEgens generally decreases with the bathochromic shift of emission wavelength has become significant in the NIR-II region. Tang et al. [52] have synthesized a series of NIR-II emissive fluorophores, whose emissions are all located in the NIR-II region with the presence of common solvents (including PhMe, DCM, CHCl_3_, THF, and DMF), while the emission intensities are relatively inferior for FLI. On the basis of the “energy gap law”, the situation above can be attributed to the ascendancy of nonradiative decay pathways when the electronic bandgap decreases, and these inherent features endow the NIR-II fluorophores with the intrinsic superiority in PAI-guided PTT, because both PAI and PTT are closely associated with the nonradiative decay [53,54].

In terms of PAI, it relies on the signal of phonons generated by the light irradiation, exceeding the traditional optical diffusion limit caused by photons after light excitation, which endows it the ability to provide higher spatial resolution [55,56,57,58] and penetrate deeper depths as high as 11 cm in NIR-II region [59,60]. More specifically, photons are converted into localized heat that induces transient thermoelastic expansion and wideband acoustic waves in the process of PAI, according to which the process of photo-to-thermo transitions is involved [61,62,63]. Therefore, the nonradiative decay pathway is closely related to the photothermal conversion property, and NIR-II fluorophores exhibit good potential for PAI.

As for the PTT process, the NIR-II fluorophores demonstrate relatively better photothermal conversion efficiency, compared with those emissions within visible spectroscopy, whose temperature variation generated by photothermal effect reaches merely 13 °C [64]. Thus, the strategy to enhance nonradiative decay, which significantly improves photothermal conversion efficiency to achieve PAI-diagnosis-guided PTT, is another appealing approach of the utilization of NIR-II fluorophores in the phototheranostic field.

Inspired by the inherent superiorities mentioned above, Tang et al. put forward a strategy to boost nonradiative decay so as to elevate the photothermal conversion efficiency using reverse thinking of the AIE process, aiming to maximize molecular motions in the aggregated state to enhance heat transitions through extending the side chain length or adding twisted groups, among which the studies of Liu et al. [52] have unprecedentedly integrated the superiorities of reversed AIE and dark TICT to achieve improved photothermal conversion, which can be described as “adjusting TICT in aggregates for boosting photothermal properties”.

**Figure 4:**
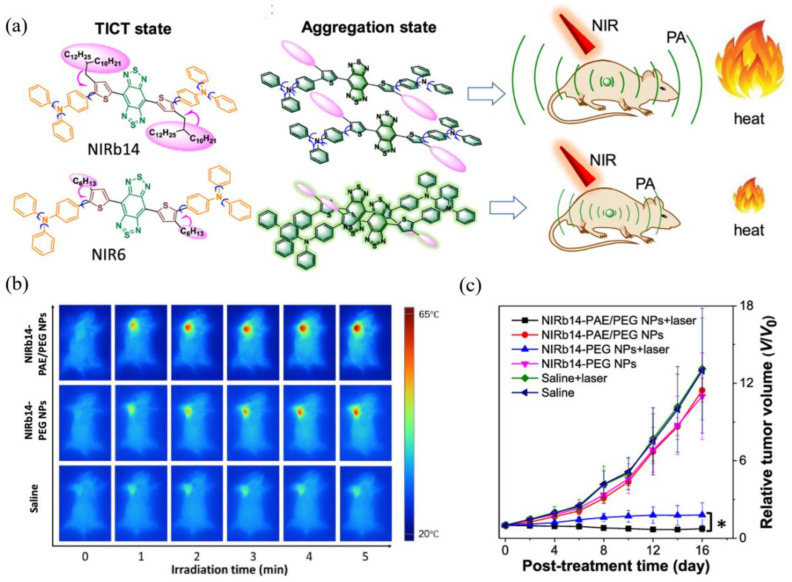
(**a**) Molecular structure and the schematic illustration of the NIR-II fluorophores TICT state in solution, in aggregation state, and the scheme for PAI-guided PTT; (**b**) infrared radiation thermal images of mice with 4T1 tumor under 808 nm laser irradiation; (**c**) tumor growth curves of different treatment groups after 16 days. *: *V/V_0_* < 0.05 comparing “NIRb14-PAE/PEG NPs + laser” and “NIRb14-PEG NPs + laser” groups. Reprinted with permission from Ref. [52]. Copyright 2019, American Chemical Society.

In this case, bulky alkyl chains were introduced into the planar D–A–D skeleton with molecular rotors as the branches (Figure 4a), from which a series of NIR-II fluorophores with different substituent groups were synthesized including NIRb14, NIRb10, NIRb6, and NIR6, where typical TICT and AIE properties were manifested. Structurally, only when the branched alkyl chains’ structure is placed in the second carbon of thiophene can the suitable steric hindrance be provided, avoiding the overwhelming hindrance that leads to intense fluorescence. NIRb14 exhibited the most outstanding photothermal conversion efficiency than the other branched molecules and the widely used golden nanorods, due to the larger bulky chains serving as shielding units that limited intermolecular interactions and conserved intramolecular motions in the aggregated state. PAE-b-PCL and PEG-b-PCL were employed to integrate NIRb14 into mixed-shell NPs (named NIRb14-PAE/PEG NPs) via nanoprecipitation method with the intention of prolonging in vivo blood circulation time and enhancing accumulation in the tumor region, since poly(β-amino esters) (PAE) is able to respond to the cancer tissue [65], and polyethylene glycol (PEG) has excellent biocompatibility. Subsequently, in thermal imaging and in vitro experiment, NIRb14-PAE/PEG NPs exhibited desirable PAI abilities and noteworthy charge conversion features in response to the acidic tumor microenvironment, as well as superior PTT performance, which motivated further investigations of in vivo PAI-guided PTT capability by using the xenograft 4T1 tumor mouse model. As indicated in Figure 4b, the thermal imaging of injected mice exhibited significant temperature variation (∆_T_ = 29 °C) at the tumor region after irradiation, in contrast to the negligible results observed from other control groups. Furthermore, by monitoring the tumor volumes during 16 days, the in vivo antitumor efficacies were examined, the results of which are illustrated in Figure 4c. Compared with those of control groups that failed in suppressing the tumor growth, in the presence of both NIRb14-PAE/PEG NPs and laser, the average tumor volume after treatment was much smaller than the initial size, demonstrating the most preeminent antitumor efficacy and great potential of PAI-guided PTT for clinical applications. This study introduces an ingenious molecular design tactic for constructing a photothermal conversion-boosted NIR-II phototheranostics by means of the stabilization of the dark TICT state or the restriction of radiative decay, in addition to demonstrating its potential in cancer diagnosis and therapy by PAI-guided PTT model.

## 4. Multimodal Imaging-Guided Synergistic Therapy

Although AIEgens displayed great potential in FLI/PAI-guided PTT, difficulties are still remained in realizing the optimal treatments via one-to-one modality. For instance, the imaging information with both favorable sensitivity and penetration depth is not able to be obtained by a single imaging modality [58,66,67,68,69], and it is also burdensome to achieve satisfactory treatment via PDT or PTT alone, which is attributed to hypoxic tumor microenvironment for PDT and heat shock effect in PTT [70,71]. Constructing multimodality phototheranostic platforms is a smart strategy that is able to achieve “1 + 1 > 2” to solve these problems, which can afford precise diagnosis and efficacious therapy via combining different kinds of imaging technologies with therapy methods, and has induced great interest recently. However, it is a challenging task because keeping the equilibrium between radiative and nonradiative decays is intractable for conventional materials, which is crucial to building up a versatile phototheranostic system with favorable fluorescent and photothermal properties concurrently. By virtue of affluent free-motioned molecular rotators or vibrators in structure, the photophysical properties of AIEgens could be manipulated easily by boosting or inhibiting intramolecular motions [16,20,72]. Furthermore, endowing AIEgens with twisted conformations could lead to relatively loose packing in the aggregated state through tactful molecular regulation, which is beneficial to balance radiative and nonradiative decays. In addition, considering the superior features of NIR-II imaging mentioned at the beginning of this review, we believe that constructing multimodality theranostic systems based on NIR-II AIEgens is a win–win integration [73].

### 4.1. NIR-II FLI-Guided PDT–PTT Synergistic Therapy

**Figure 5:**
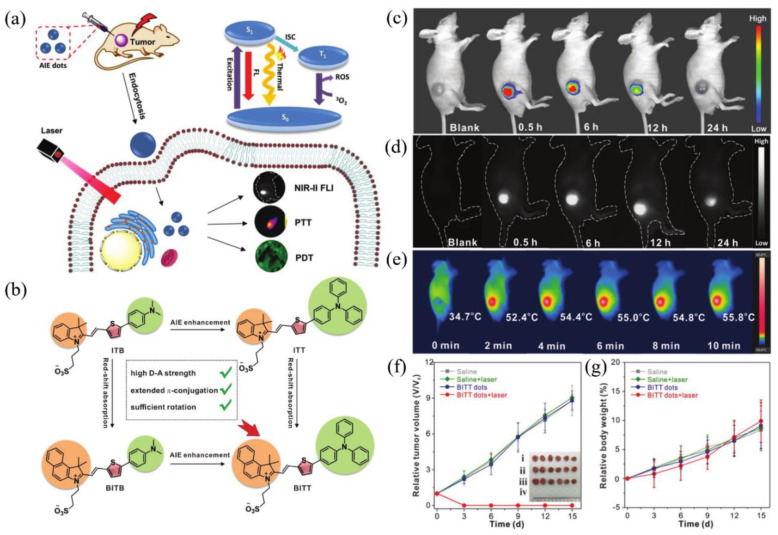
(**a**) Diagram of NIR-II FLI-guided PDT–PTT synergistic therapy for tumor-bearing mice with BITT NPs; (**b**) principle for designing BITT; (**c**) NIR-I FLI and (**d**) NIR-II FLI of tumor-bearing mice after administration of BITT NPs at different monitoring times; (**e**) IR imaging of tumor-bearing mice under laser irradiation at 12 h postinjection of BITT NPs; (**f**) growth curves of tumors with various treatments. Inset: photograph of tumors harvested from the mice at day 15 after different treatment; (**g**) body weight curves of tumor-bearing mice after different treatments at day 15. Reprinted with permission from Ref. [75]. Copyright 2021, Wiley-VCH.

It is hard to achieve satisfactory treatment via PDT and PTT alone, as described in the previous section, and the combination of them is regarded as a groundbreaking strategy to overcome respective shortcomings and realize boosted synergistic therapeutic outcomes [8,11,74] because PTT could heighten the oxygen concentration by increasing the flow rate of blood to strengthen the PDT effect, thus promoting the elimination of heat-resistant tumor in PTT reversely. Recently, a novel zwitterion-type AIEgen was facilely synthesized, which could afford effective NIR-II FLI-guided synergistic PDT-PTT [75]. In this study, the author designed a series of zwitterionic compounds (BITT, BITB, ITT, and ITB in Figure 5b). The extra benzene ring in BITT would elongate π conjugation and increase D–A strength, resulting in a longer absorption wavelength than ITT in ethanol solution and admirable ROS generation capacity [76]. Meanwhile, compared with BITB, the AIE performance of BITT would be dramatically strengthened due to the introduction of triphenylamine moiety. The alkyl chain with the sulfonic acid group and twisted TPA moiety would enlarge the intermolecular distance, which is beneficial to restrain fluorescence quenching by intermolecular π–π stacking in aggregated state and lead to relatively loose intermolecular packing (reserve partial intramolecular motion), endowing BITT with both intense NIR-II fluorescence and high photothermal conversion capability through keeping the balance between radiative and nonradiative decays. Moreover, BITT would also exhibit superior biocompatibility owing to the zwitterionic structure [77]. Photophysical experiments demonstrated that BITT NPs in aqueous solution possessed a long emission wavelength peak at 810 nm, with the tail partially located in the NIR-II region, high photothermal conversion efficiency (35.76%), excellent ROS generation capability, as well as high photostability and photothermal stability.

Motivated by prominent photophysical properties within BITT NPs, in vivo imaging and therapy performance of BITT NPs for 4T1 tumor-bearing nude mice were estimated subsequently. As shown in Figure 5c,d, compared with NIR-I FLI, an intense and durable NIR-II fluorescence signal was observed from 0.5 to 24 h postinjection. After being exposed to laser irradiation for only 2 min, the temperature of the tumor region raised from 34.7 to 52.4 °C (Figure 5c), which was competent to suppress tumor tissue efficaciously. As indicated in Figure 5f,g, the tumors were completely eliminated without any recidivation via synergistic PDT–PTT therapy in the presence of BITT NPs and laser irradiation, and no obvious body weight loss was observed in four groups, demonstrating excellent tumoricidal capability and biocompatibility. This study thus provided a new strategy to construct a NIR-II AIEgen with both FLI imaging and PTT–PDT therapy.

### 4.2. NIR-II FLI–PAI–PTI Trimodal-Guided Synergistic PDT–PTT

**Figure 6:**
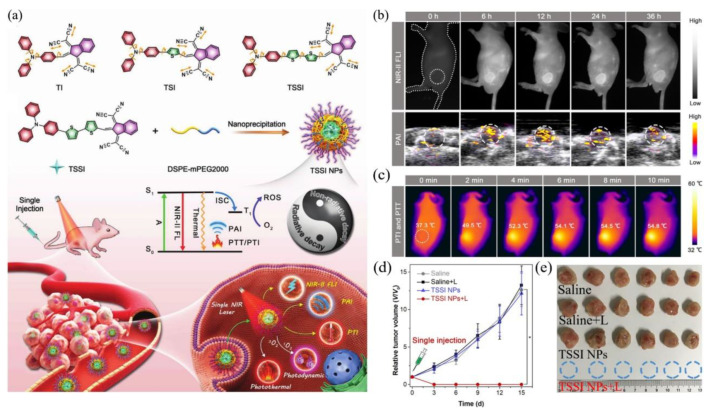
(**a**) Diagram of all-around AIE molecular structures, nanoparticles fabricating, and multimodal phototheranostic system; (**b**) NIR-II FLI (upper) and PAI (lower) for tumor-bearing mice from 0 h to 36 h postinjection of TSSI NPs; (**c**) PTI and PTT for tumor-bearing mice at 12 h postinjection of TSSI NPs upon laser irradiation; (**d**) tumor growth curves of mice with various treatments; (**e**) photograph of 4T1 tumors harvested from the mice at day 15 after different treatment. Reprinted with permission from Ref. [83]. Copyright 2020, Wiley-VCH.

Considering the complementary advantages of FLI, PAI, and PTI, as well as boosted therapeutic effect of PDT and PTT, constructing single-component theranostic platforms that can afford all these phototheranostic modalities simultaneously would be extremely important [78,79,80,81,82]. Motivated by the superiorities of NIR-II AIEgens mentioned before, a number of remarkable multifunctional phototheranostic platforms have been developed upon NIR-II AIEgens. A simple and one-for-all phototheranostic platform with NIR-II AIE characteristics was reported [83], which could afford NIR-II FLI–PAI–PTI trimodal-imaging-guided synergistic PDT–PTT. As illustrated in Figure 6a, three novel AIE compounds composed of 1,3-bis (dicyanomethylidene) indane, thiophene and triphenylamine were prepared. The twisted conformation of TPA would extend the intermolecular distance and lead to loosened packing in an aggregated state, which is helpful to retain intramolecular motions partially, and the stretching vibrations of carbon–nitrogen bonds would maintain considerable frequency even in the aggregated state, thus strengthening the heat generation efficiency of AIEgens. Benefiting from the increasing number of thiophene units, compared with TI and TSI, TSSI, as well as TSSI NPs fabricated with DSPE-mPEG2000, would exhibit more admirable properties for phototheranostics since the addition of thiophene moiety could enhance D–A strength and the capacity of ROS generation and also enlarge the distance of donor and acceptor within the AIEgens, further boosting intramolecular motions. As expected, TSSI NPs exhibited a maximum emission near 1000 nm, indicating remarkable properties for NIR-II imaging, and they also possessed superior ROS and heat generation capabilities, both of which confirmed the perfect equilibrium between radiative and nonradiative decays. Furthermore, TSSI NPs were utilized to implement in vivo experiments for tumor-bearing mice. Obvious fluorescence signals (Figure 6b, upper) were observed from 6 to 36 h and reached a maximum at 12 h after injection in NIR-II FLI. Surprisingly, the time-dependent tendency of PA intensity (Figure 6b, lower) was nearly unanimous with NIR-II FLI outcomes for the same tumor model. In in vivo experiments of PTI and PTT (Figure 6c), upon NIR irradiation for only 10 min, the temperature of the tumor region raised from 37.3 °C to 54.8 °C, and insignificant temperature variation was observed in the normal tissue. As shown in Figure 6d, e, the tumor tissues of mice were obliterated completely upon both TSSI NPs and NIR irradiation through only one injection and irradiation. This study offered a smart tactic to construct one-for-all AIEgen, but it also demonstrated its great potential in multimodality theranostics.

Shortly thereafter, several outstanding single-component multimodal theranostic platforms were constructed tactfully. A novel type of theranostic AIEgen was designed under the guidance of a similar principle as the above-mentioned [84]. In this study, three AIE compounds (TAM, TSAM, TSSAM) with none, one, two thiophenes were prepared, respectively. As expected, TSSAM possessed NIR-II emission, high photothermal conversion efficiency, and ROS generation capability, as well as outstanding photostability and photothermal stability. TSSAM NPs exhibited favorable accordance and intratumor retention capability for in vivo NIR-II FLI and PAI, where the signal intensities of NIRI-I FLI and PAI reached a plateau at 12 h postinjection and remained durable at 24 h postinjection. Upon laser irradiation for only 2 min, the temperature of the tumor region increased from 37.1 to 57.6 °C. In the presence of TSSAM NPs and laser irradiation, all tumors were eradicated on day 3. Later, Wen et al. [85] also designed four novel NIR-emissive AIEgens. Benefiting from the highly bright emission of the tail located in the NIR-II region in the aggregated state, photothermal conversion efficiency, and high ROS generation capability of TTT-4, in vivo multimodal imaging and therapy performance of TTT-4 dots was evaluated. Significant fluorescence signals were detected in both NIR-I and NIR-II FLI from 1 to 12 h postinjection at the tumor sites, as well as intense PA signals captured by the PAI system. The temperature of the tumor region raised from 37.3 to 55 °C upon laser irradiation for only 3 min, and the results of in vivo synergistic phototherapeutic experiments illustrated that tumor tissue was completely eradicated after 15-day treatment. In general, TSSAM and TTT-4 enrich the types of versatile phototheranostic AIEgens and display great potential in NIR-II FLI–PAI–PTI-guided synergistic PDT–PTT.

## 5. Conclusions

In this minireview, we summarized the existing strategies for constructing efficacious theranostic platforms based on NIR-II AIEgens and their great potential in basic studies and practical applications. Through subtle regulation of hierarchical structures at molecular and aggregated levels, respectively, the AIE and TICT properties could be manipulated, thus achieving admirable equilibrium between fluorescence and photothermal effect, as well as superior performance in NIR-II FLI-guided PTT against cancer. To further boost photothermal properties for PAI and PTT, the strategy named “reverse thinking of AIE” is proposed. With the existence of long-branched alkyl chains and molecular motors in the molecule, it displayed excellent photothermal conversion efficiency and photoacoustic effect, thus achieving remarkable antitumor efficacy in PAI-guided PTT. Furthermore, in order to overcome the inherent drawbacks in one-to-one modality, a series of versatile theranostic molecules with AIE characteristics were prepared. On account of tactful molecule design, these AIE molecules can make the best utilization of excited-state energy and keep a remarkable balance between radiative and nonradiative decays, thus exhibiting highly bright emission, prominent photothermal conversion efficiency, and efficacious ROS generation to achieve NIR-II FLI-guided PTT–PDT. Some of them even suffice to NIR-II FLI–PAI–PTI trimodal-guided synergistic PDT–PTT, and in vivo experiments revealed that these AIE NPs could afford precise tumor imaging and thorough tumor elimination outcomes.

Although reported phototheranostic systems based on NIR-II AIEgens validated great potential in both basic studies and clinical practices, there are still challenges that should be addressed. Firstly, the development of new AIEgens with a longer emission wavelength for the theranostic study is urgent, since the maximum emission wavelength of all reported theranostic AIEgens is below 1100 nm. Secondly, as a vital guideline for designing theranostic molecules, the structure–property relationship of AIEgens for theranostic applications is still obscure [7]. Thirdly, the current achievements of phototheranostics are still far from clinical applications. Although the biocompatibility of AIEgens has been demonstrated by substantial experimental data, more comprehensive research studies are in urgent need for further clinical trials, as well as investigation of their long-term safety using in vivo experiments with diverse animal models. The results of this review also call for constructing functional materials based on NIR-II AIEgens to target specific organs or in response to external stimuli.

Overall, NIR-II AIEgen is a predominant candidate to construct a phototheranostic system with superior performance in basic research and practical applications, which opens up new avenues for advanced theranostics and will realize clinical trials in the near future.

## Figures and Tables

**Table 1:** The chemical structures and essential photophysical properties of NIR-II AIEgens.

Name	Chemical Structure	λ_Abs_/λ_em_ (nm)	Extinction Coefficient	QY	Photothermal Conversion
BPBBT	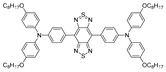	≈705/ ≈1020 (in water, NPs)	0.9 × 10^4^ M^−1^·cm^−1^ at 808 nm	0.145% ^[a]^ (NPs)	27.5% (NPs, 808 nm laser)
PBPTV	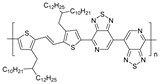	≈700/≈960 (in water, NPs)	N.A.	8.6% ^[b]^ (in DCM)	45.3% (NPs, 808 nm laser)
DTPA–BBTD	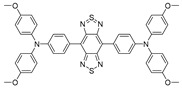	752/975 (in THF)	7.09 L·g^−1^·cm^−1^ at 753 nm	0.151% ^[a]^ (NPs)	13.2% (NPs, 660 nm laser)
NIRb14	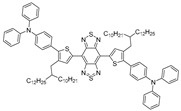	822/1090 (in THF)	N.A	N.A.	31.2% (NPs, 808 nm laser)
BITT	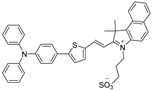	595/741 (in ethanol)	3.9 × 10^4^ M^−1^·cm^−1^	5.8% ^[b]^ (aggregate)	35.76% (660 nm laser)
TSSI	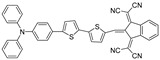	636/992 (in water, NPs)	N.A.	N.A.	46.0% (NPs, 660 nm laser)
TSSAM	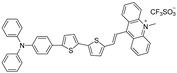	595/1022 (in DMSO)	N.A.	0.034% ^[a]^ (NPs)	40.1% (NPs, 660 nm laser)
TTT-4	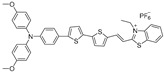	568/830 (in DMSO)	N.A.	0.8%^[b]^ (aggregate, DMSO:toluene = 19:1)	39.9% (NPs, 660 nm laser)

[a] Relative PL quantum yields of these molecules recalculated based on IR-26 = 0.05%. [b] Absolute PL quantum yields.

## Data Availability

Not applicable.
